# Why invest in early childhood?

**DOI:** 10.1590/1518-8345.0000-3253

**Published:** 2020-02-03

**Authors:** Sonia Isoyama Venancio

**Affiliations:** 1 Secretaria de Estado da Saúde de São Paulo, Instituto de Saúde, São Paulo, SP, Brazil.

**Figure:**
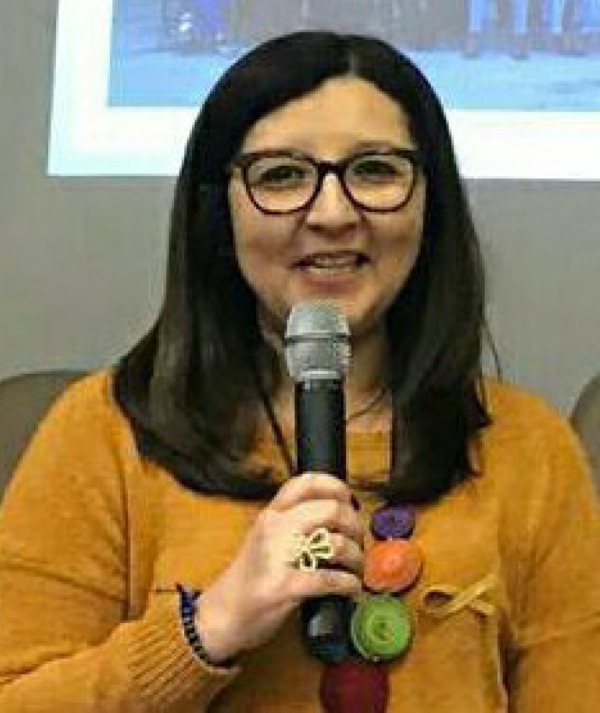

Brazil defined health as a universal right in the 1988 Federal Constitution, by creating the Brazilian Unified Health System (SUS) and, in 1990, the full protection of children by the Statute of the Child and Adolescent (ECA)^(^ 1 ^)^. Since then, children’s health has been showing significant improvement in the country. There has been a reduction in morbidity and mortality caused by immuno-preventable diseases and diarrhea, a decrease in malnutrition rates and a growing improvement in breastfeeding indicators^(^ 1 ^)^. As a result, Brazil has achieved a decrease in infant mortality (under one year) and child mortality (under five years), meeting the 2015 Millennium Development Goal (MDG) three years in advance^(^ 1 ^)^. Likewise other countries presenting child mortality reduction, aspects related to children’s well-being and their full development tend to gain relevancy.

Early childhood development (from zero to six years old in Brazil^(^ 1 ^)^) has gained increasing prominence thanks to the contribution of research in neuroscience and public policy. It is currently known that the intrauterine period and early years of life are essential for the physical, emotional and cognitive development of children. Along pregnancy and the first years of life (especially during the first thousand days), rapid brain development occurs, and also at this stage the neural circuits are formed and strengthened through stimulation and bonding relationships. Physical and emotional health, social skills, and cognitive-language skills that emerge in the early years are important prerequisites for success in school, and later in the workplace and community^(^ 2 ^)^.

Evidence indicates that investment in quality early childhood programs provides society with a high rate of return. In addition, early childhood investment is the best way to reduce inequalities, address poverty and build a social and environmentally sustainable society^(^ 3 ^)^. Despite the evidence of early childhood importance, it is estimated that over 200 million children under the age of five in low and middle-income countries do not reach their developmental potential due to exposure to environmental, biological and psychosocial risk factors^(^ 3 ^)^.

To address this problem, some initiatives have been taken worldwide. The importance of early childhood development is endorsed by the 2030 Sustainable Development Goals, and the Early Childhood Development Action Network - consisting of the United Nations Children’s Fund (UNICEF), the World Bank and the World Health Organization - who proposed the “Nurturing care model” to encourage countries to invest in intersectoral programs. According to this model, early child development care and attention should include health, nutrition, responsive care, early childhood learning, protection and safety^(^ 4 ^)^.

The number of countries with intersectoral policies for early childhood development has increased from 7 in 2000 to 68 in 2014, of which 45% were low and middle-income countries^(^ 3 ^)^. Following the global trend, investment in Brazil is increasing in promoting the development of this age group through the implementation of federal programs such as *Brasil Carinhoso* (Affectionate Brazil) and *Criança Feliz* (Happy Child) along with other state and local initiatives^(^ 5 ^)^. An important step towards strengthening this agenda at national level is the establishment of the *Marco Legal da Primeira Infância* (Early Childhood Legal Framework), which sets principles and guidelines for public policies formulation and implementation for early childhood, considering the specificity and relevance of the early years of life both in child and human development^(^ 1 ^)^.

In the health area, child development promotion was also emphasized in the *Política Nacional de Atenção Integral à Saúde da Criança* (PNAISC - National Policy for Integral Child Health Care), published in 2015. In its third strategic action axis, this policy highlights the importance of surveillance and encouragement for “full growth and development of children, especially the ‘Early Childhood Development (DPI)’, by Primary Health Care, as directed by the Child Health Handbook, which includes support actions to strengthen family bonds^(^ 1 ^)^.

Thus, there is an increasing interest in the implementation of policies aimed at early childhood in Brazil, with the commitment of federal, state and municipal managers, as well as the engagement of civil society. In this context, the role of health professionals, through early contact with children and their families is to contribute to the promotion of health, proper nutrition, strengthening of bonds, and early and appropriate stimulation, which is fundamental to ensure that all children reach their full developmental potential.
